# Assessing the Performance of GPS Precise Point Positioning Under Different Geomagnetic Storm Conditions during Solar Cycle 24

**DOI:** 10.3390/s18061784

**Published:** 2018-06-01

**Authors:** Xiaomin Luo, Shengfeng Gu, Yidong Lou, Chao Xiong, Biyan Chen, Xueyuan Jin

**Affiliations:** 1 GNSS Research Centre, Wuhan University, Luoyu Road 129, Wuhan 430079, China; xmluo@whu.edu.cn (L.X.); ydlou@whu.edu.cn (L.Y.); 2 GFZ German Research Centre for Geosciences, Telegrafenberg, 14473 Potsdam, Germany; bear@gfz-potsdam.de; 3 School of Geosciences and Info-Physics, Central South University, Changsha 410083, China; yeary124@csu.edu.cn; 4 School of Geodesy and Geomatics, Wuhan University, Luoyu Road 129, Wuhan 430079, China; xueyuan.jin@whu.edu.cn

**Keywords:** geomagnetic storms, precise point positioning, ionospheric irregularities, rate of TEC index

## Abstract

The geomagnetic storm, which is an abnormal space weather phenomenon, can sometimes severely affect GPS signal propagation, thereby impacting the performance of GPS precise point positioning (PPP). However, the investigation of GPS PPP accuracy over the global scale under different geomagnetic storm conditions is very limited. This paper for the first time presents the performance of GPS dual-frequency (DF) and single-frequency (SF) PPP under moderate, intense, and super storms conditions during solar cycle 24 using a large data set collected from about 500 international GNSS services (IGS) stations. The global root mean square (RMS) maps of GPS PPP results show that stations with degraded performance are mainly distributed at high-latitude, and the degradation level generally depends on the storm intensity. The three-dimensional (3D) RMS of GPS DF PPP for high-latitude during moderate, intense, and super storms are 0.393 m, 0.680 m and 1.051 m, respectively, with respect to only 0.163 m on quiet day. RMS errors of mid- and low-latitudes show less dependence on the storm intensities, with values less than 0.320 m, compared to 0.153 m on quiet day. Compared with DF PPP, the performance of GPS SF PPP is inferior regardless of quiet or disturbed conditions. The degraded performance of GPS positioning during geomagnetic storms is attributed to the increased ionospheric disturbances, which have been confirmed by our global rate of TEC index (ROTI) maps. Ionospheric disturbances not only lead to the deteriorated ionospheric correction but also to the frequent cycle-slip occurrence. Statistical results show that, compared with that on quiet day, the increased cycle-slip occurrence are 13.04%, 56.52%, and 69.57% under moderate, intense, and super storms conditions, respectively.

## 1. Introduction

Geomagnetic storms, i.e., large-scale disturbances in the Earth’s near-space environment, are caused by the enhanced solar wind and its interaction with the magnetosphere–ionosphere–thermosphere system. According to the Dst index derived from near-equatorial geomagnetic measurement, the principal feature of a geomagnetic storm represents an obvious decrease of the horizontal intensity of Earth’s magnetic field followed by a recovery [1]. The disturbance that drives geomagnetic storm can be due to the occurrence of coronal mass ejections (CMEs) on the Sun and the associated interplanetary shock waves or corotating interaction region (CIR) produced by high-speed solar wind streams in the interplanetary medium [1,2]. Generally, a geomagnetic storm consists of three periods, i.e., initial phase, main phase, and recovery phase [3].

A geomagnetic storm is characterized by an increased spatial decorrelation of ionosphere range delays and scintillation effects at both high- and low-latitudes [4]. During the geomagnetic storm, abundant energetic particles inject into the Earth’s magnetosphere. Some of them follow the terrestrial magnetic field and then precipitate into the ionospheric altitude at high-latitude, resulting in steep ionospheric density gradients and irregularities [5]. The ionospheric irregularities at high-latitude including polar cap patches and auroral blobs can both cause rapid phase and amplitude fluctuations of the trans-ionospheric radio signals, known as ionospheric scintillation [6]. During a geomagnetic storm, the occurrence of equatorial irregularities including equatorial spread-F (ESF) and plasma bubbles (EPBs) is quite complicated since it is influenced by many factors such as the existence of prompt penetration, disturbance dynamo electric fields, and small increase of geomagnetic activity level, which are all known affect the vertical plasma drifts and thus dominate the Rayleigh-Taylor linear growth rate [7]. In addition, the ESF and EPBs triggered by the geomagnetic storm may also depend on the maximum dDst/dt determined local time sector [8]. The occurrence equatorial irregularities during geomagnetic storms can induce the equatorial ionospheric scintillation. To GNSS users, ionospheric scintillation possibly leads to the occurrence of cycle-slip and even loss of signal tracking, degenerating the GNSS positioning and navigation accuracy [9,10,11].

Over the past decades, many researchers have reported the adverse effects of geomagnetic storm on GPS positioning. Usually, network real-time kinematic (RTK) can achieve cm-level accuracy over distances within ~100 km [12]. However, this level of accuracy would be significantly affected by the geomagnetic storm [13]. Due to the fast ionospheric error decorrelation in the super storm on 29 October 2003, the success ratio of instantaneous ambiguity resolution of RTK reduced to only 31% compared to 94% on quiet day [14]. As a result, the RTK positioning error in the up component exceeded 0.5 m for a 121 km baseline during this storm. Similar results by Bergeot et al. [15] also indicated that, during the geomagnetic storm period of 30 October 2003, the position repeatability of the kinematic GPS positioning in northern Europe can reach 12.8 cm, 8.1 cm and 26.1 cm for the north, east, and up components, respectively, while it was better than 2.5 cm under normal ionospheric condition. Except for ionospheric disturbances, the degradation of RTK positioning is also affected by the baseline orientation during geomagnetic storm period. The maximum and standard deviation values of positioning error for baselines with a north–south orientation are larger than those baselines with east–west orientation [16]. More recently, focusing on Norway region, a detailed analysis of network RTK performance and phase scintillation caused by the super storm on St. Patrick’s Day (17 March) 2015 was presented by Jacobsen and Andalsvik [17]. The simple function relationship between vertical positioning error and phase scintillation index, known as the rate of total electron content (TEC) index (ROTI), was shown in their work. Results indicated that the positioning errors increased exponentially with the rise of ROTI values.

With respect to the studies on network RTK technique, the effects of geomagnetic storm on precise point positioning (PPP) have only received limited attention. PPP is a stand-alone positioning technique, which normally can achieve positioning accuracy of dm-level to cm-level using undifferenced dual-frequency observations with precise satellite orbit and clock products [18,19]. Under the same geomagnetic storm condition, Jacobsen and Andalsvik [17] found that the performance of PPP is always better than that of RTK at all ionospheric disturbance levels in Norway region. However, only three stations within limited latitude at 55°–70° N were considered in their study. To provide a more representative investigation, in this work we collect data from about 500 International GNSS services (IGS) stations and present a comprehensive assessment on the performance of both dual-frequency (DF) and single-frequency (SF) PPP under different intensities of geomagnetic storm during solar cycle 24. This article is organized as follows: Section 2 describes the data and methodology in details. Section 3 presents GPS DF and SF PPP solutions under three typical geomagnetic storms conditions, i.e., moderate, intense, and super storms, during solar cycle 24 from 1 January 2011 to 31 December 2017. Furthermore, the corresponding analyses for the experiments are also shown in Section 3. Finally, the discussion and conclusion are given in Section 4 and Section 5, respectively.

## 2. Data and Methodology

This section describes the data sources, including geomagnetic activity, solar activity, global TEC maps, and GPS measurements, used in our study. In addition, the data processing models and strategies used to perform GPS DF and SF PPP are presented in this section.

### 2.1. Data Sources

The Dst index is a good quantitative measure of the intensity of the geomagnetic storm [20]. In this work, we focus only on the geomagnetic storm with minimum Dst (Dst_min_) ≤ −50 nT, which is commonly adopted as the threshold by many past studies [21,22]. Further, if a period of high activity showed multiple Dst ≤ −50 nT, we arbitrarily treat them as a single storm event if the minima were separated by less than 24 h, rather than define each minimum as a separate storm [23]. Depending on Dst index, the storms are classified as moderate (−100 nT < Dst_min_ ≤ −50 nT), intense (−200 nT < Dst_min_ ≤ −100 nT), and super storm (Dst_min_ ≤ −200 nT).

**Figure 1:**
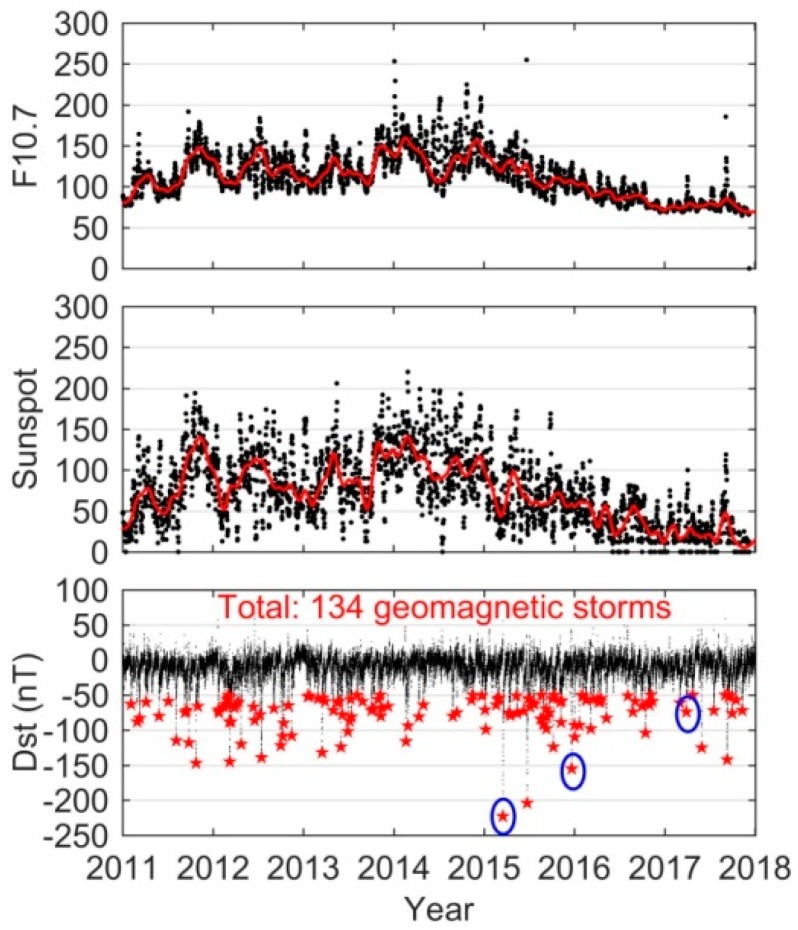
The variations of solar flux index F10.7, sunspot number and the geomagnetic index Dst in the solar cycle 24. The red lines represent the corresponding smoothed data and the red stars represent the geomagnetic storms.

Figure 1 presents the variability of solar flux index F10.7, daily sunspot number and the hourly Dst distribution during solar cycle 24 from 1 January 2011 to 31 December 2017. The red lines in the top two panels represent the corresponding smoothed data. The red stars in the bottom panel represent the geomagnetic storms. Statistics show that there are total 134 geomagnetic storms, including 113 moderate storms, 18 intense storms, and 2 super storms, during solar cycle 24 from 1 January 2011 to 31 December 2017. To fully assess the performance of GPS PPP under geomagnetic storms conditions, we select three typical storms data to perform the experiments, which are highlight in blue ellipses shown in the third panel of the figure. They are moderate storm on March 27, 2017 with Dst_min_ = −74 nT, intense storm on 20 December 2015 with Dst_min_ = −155 nT, and super storm on 17 March 2015 with Dst_min_ = −233 nT.

**Figure 2:**
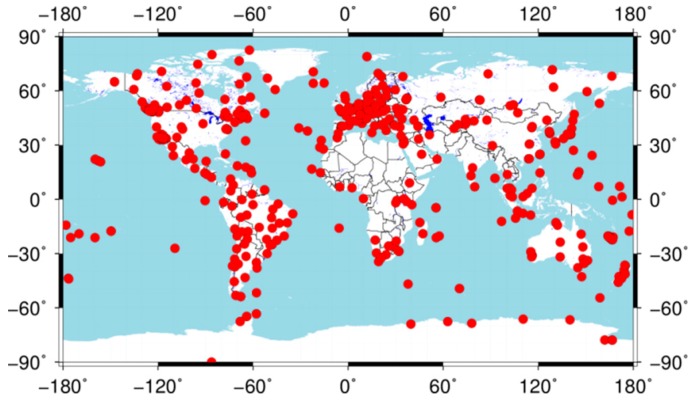
Geographical distribution of the 492 IGS stations.

In this work, the GPS dual-frequency data provided by the IGS are used to investigate the effects of storms with different intensities on GPS PPP solutions (ftp://cddis.gsfc.nasa.gov/). There are about 500 continuous operation tracking stations distributed as shown in Figure 2. Besides, the global TEC maps with a time resolution of 5 min derived from the worldwide GNSS stations are used to analyze the ionospheric response to various geomagnetic storms. The TEC map data can be freely accessible from the CEDAR Madrigal database (http://cedar.openmadrigal.org/ftp/).

### 2.2. GPS PPP Model

The raw observables of dual-frequency GPS pseudorange and carrier phase between a receiver “*r*” and a satellite “*s*” are generally expressed as below [24]:

(1) $$P_{r,f}^{s}=\rho_{r}^{s}+t_{r}-t^{s}+\alpha_{r}^{s}\cdot T_{z}+b_{r,f}-b_{f}^{s}+\beta_{f}\cdot I_{r}^{s}+\varepsilon_{P}$$

(2) $$\Phi_{r,f}^{s}=\rho_{r}^{s}+t_{r}-t^{s}+\alpha_{r}^{s}\cdot T_{z}+b_{r,f}-b_{f}^{s}-\beta_{f}\cdot I_{r}^{s}-\lambda_{f}\cdot \left(N_{r,f}^{s}-\varphi \right)+\varepsilon_{\Phi }$$

where $P_{r,f}^{s}$ and $\Phi_{r,f}^{s}$ are the pseudorange and carrier phase on frequency f in meters; ρ is the geometric range in meters; $t_{r}$ and $t^{s}$ are the receiver and satellite clock offset; $T_{z}$ is the zenith tropospheric delay; α is the mapping function; $b_{r}$ and $b^{s}$ are the frequency dependent signal delay for receiver and satellite; I is the line-of-sight TEC with the frequency dependent factor $\beta_{f}$ = 40.3/$f^{2}$; λ is the wavelength; N is the float ambiguity and φ is the phase windup error in cycle; and $\varepsilon_{P}$ and $\varepsilon_{\Phi }$ are the measurement noise of pseudorange and carrier phase, respectively.

In this study, we adopted undifferenced PPP model with raw GPS measurements instead of ionospheric-free PPP model. Compared with the latter one, in the undifferenced model, individual signals of each frequency are treated as independent observables, thus avoiding noise amplification in the linear combinations. For the ionospheric delay in DF and SF PPP models, a priori ionospheric model with proper constraints is utilized as [25]:

(3) $$I_{r}^{s}=\gamma_{r}^{s}\cdot I{\left(z\right)}_{r}^{s}$$

(4) $$I{\left(z\right)}_{r}^{s}=a_{0}+a_{1}dL+a_{2}dL^{2}+a_{3}dB+a_{4}dB^{2}+r_{r}^{s}$$

(5) $$\tilde{I}{\left(z\right)}_{r}^{s}=a_{0}+a_{1}dL+a_{2}dL^{2}+a_{3}dB+a_{4}dB^{2}+r_{r}^{s}+\varepsilon_{\tilde{I}{\left(z\right)}_{r}^{s}}$$

where $I{\left(z\right)}_{r}^{s}$ is the vertical TEC of the ionospheric pierce point (IPP); γ denotes the ionosphere mapping function; $a_{i}$ (*i* = 0, 1, 2, 3, 4) are the coefficients used to describe the deterministic behavior of the ionospheric delay; dL and dB are the longitude and latitude difference between IPP and the approximate location of station; the scalar field $r_{r}^{s}$ represents the stochastic component from a second-order stationary process; and $\tilde{I}{\left(z\right)}_{r}^{s}$ is the vertical ionospheric delay correction interpolated from global ionospheric map (GIM) with corresponding noise $\varepsilon_{\tilde{I}{\left(z\right)}_{r}^{s}}$.

In the data processing, all GPS observations have a sampling interval of 30 s, and the satellite elevation mask angle is set to 10°. GPS precise satellite orbit and clock products provided by IGS are adopted for PPP processing. The data processing models and strategies for GPS DF and SF PPP are summarized in Table 1. Note that in this study the experiments and results of DF PPP are introduced first and those of SF PPP are shown next since the DF receivers are more common used in real surveying applications. In addition, the following experiments only focus on the kinematic PPP solutions since geomagnetic storms have no obvious influence on static PPP with a daily processing.

## 3. Results and Analyses

**Figure 3:**
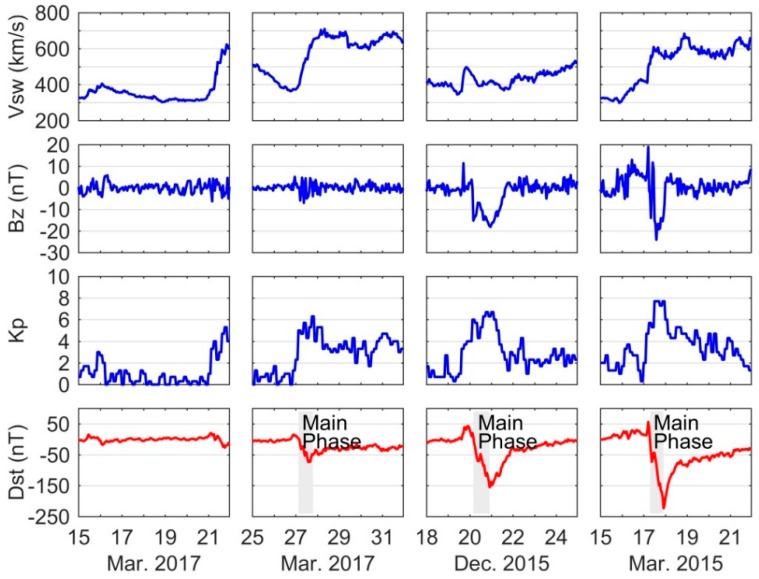
The variations of solar wind speed Vsw, IMF B_Z_, Kp index and geomagnetic index Dst during the quiet, moderate, intense, and super storm periods.

The variations of solar wind velocity (Vsw), IMF Bz component, Kp index, and Dst index during three typical storms are shown in Figure 3. For comparison, the observations on the quiet day of 17 March 2017 are also given in the figure. Obviously, the time series of Vsw and Bz show a smooth variation on 17 March 2017 due to the quiet solar activity. For the moderate storm, the Vsw is found to gradually increase on 27 March 2017, and the Bz shows rapidly variations but the magnitude is small within ±8 nT. The geomagnetic index Dst shows a decrease until 14:00 coordinated universal time (UTC) when arriving at the minimum of −74 nT. For the intense and super storms, the Vsw shows quick increases at the beginning of the storm sudden commencement (SSC) time. The IMF Bz components for the two storms are also found being rapid fluctuations when the solar winds are very dynamic. Most importantly, the duration time of negative Bz for the intense and super storms can reach several hours, which are much longer than that of moderate storm; moreover, the larger magnitude of negative Bz around −15 nT can be clearly seen in those two great storms. That is why those storms are much stronger than the moderate storm of 27 March 2017. From the Kp index panels, we can find that the values of Kp in the main phase of intense and super storms are in the range of 6 to 8, which are generally larger than those of moderate storm as the range of 4 to 6. As a response to the solar wind and IMF fluctuations, the disturbances are seen in the geomagnetic index Dst with the minimum values as −155 nT on 20 December 2015 and −233 nT on 17 March 2015.

**Figure 4:**
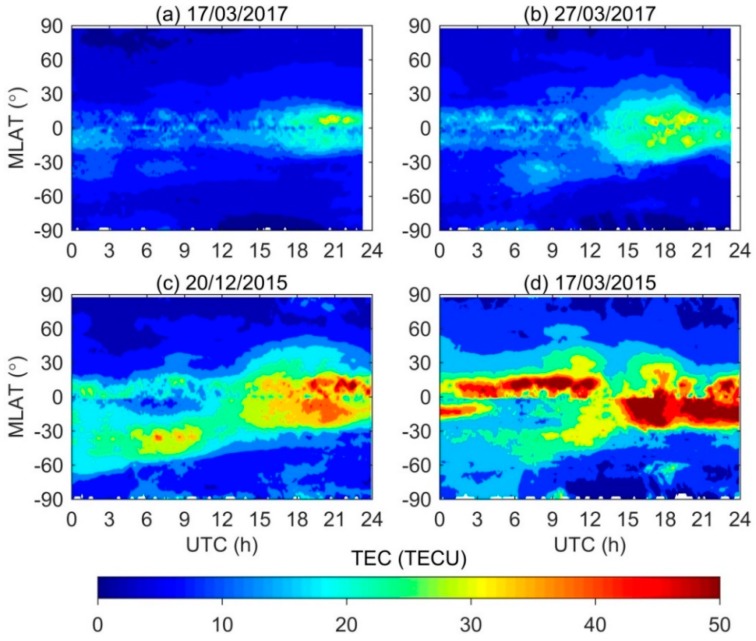
The temporal TEC variations from 90° S to 90° N MLAT on: (**a**) 17 March 2017 as the quiet day reference; (**b**) 27 March 2017 as the moderate storm; (**c**) 20 December 2015 as the intense storm; and (**d**) 17 March 2015 as the super storm, respectively.

To analyze the ionospheric response to geomagnetic storms with different intensities, the corresponding magnetic latitude (MLAT) versus UTC of TEC maps are presented in Figure 4. The TEC measurements are averaged over 5 min in UTC and 2.5° MLAT over all longitudes. Note that no data of TEC map during 23:17–24:00 UTC on 17 and 27 March 2017, so the corresponding regions are blank in Figure 4a,b. On the quiet day, large values of TEC are found in low-latitude, and the peaks are found in the equatorial ionization anomaly (EIA) regions around ±15° MLAT during 18:00–23:17 UTC. During the moderate geomagnetic storm, the crests of EIA further extend to about ±17° MLAT and the larger magnitude TEC are observed during 15:00–23:17 UTC when the western hemisphere was on dayside, which is the same as on the quiet day. It is easy to understand that the daytime TEC is larger than that on the nightside. During the intense geomagnetic disturbed condition, the crests of EIA can reach around ±22° MLAT, spreading toward mid-latitude. In addition, their TEC during 18:00–22:00 UTC can exceed 40 TECU. For the super storm on 17 March 2015, TEC values of more than 40 TECU are easily observed around ±30° MLAT on the whole day. In Figure 4, we can find that with the increase of geomagnetic storms intensity, the EIA also expands far from their regular position of ±15° MLAT and the TEC values also increase to a larger magnitude as 40–50 TECU.

**Figure 5:**
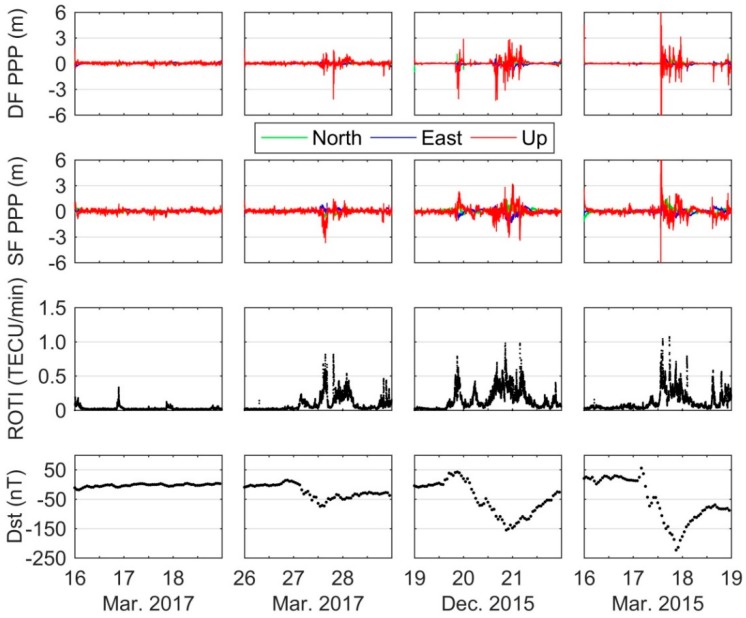
Time series of positioning errors of DF and SF PPP, ROTI, and Dst index for the KIRU station during the quiet, moderate, intense, and super storms period.

Figure 5 shows the positioning errors of GPS DF and SF PPP in the north, east and up components for the KIRU station (67°51′ N, 20°58′ E; geomagnetic: 65°15′ N, 115°20′ W) during each storm within three days. Positioning errors on 16–18 March 2017 under normal ionospheric condition are also presented in the figure for comparison. The ionospheric irregularities index as ROTI and geomagnetic index as Dst are given in the bottom panels of Figure 5. Under quiet condition of 17 March 2017, the positioning results show smooth variations with 99.2% and 92.7% of errors in the range of ±0.3 m for DF and SF PPP. However, during geomagnetic storm periods, the time series of positioning errors vary significantly especially in the up component regardless of DF or SF PPP. For instance, the positions of DF PPP during 13:00–23:00 on 17 March 2015 show outliers reaching 12.293 m, 3.693 m and 22.009 m in the north, east and up components, respectively. The detailed statistics of DF and SF PPP results are presented in Table 2. In Figure 5 and Table 2, we can find that stronger intensity geomagnetic storms generally cause more significant degradation of GPS PPP.

From the ROTI panels, it is observed that under geomagnetic storms conditions the time series of ROTI fluctuate significantly during the main phase as well as the recovery phase period, and the values can increase to around 1.0 TECU/min. Conversely, most of them in the quiet ionospheric condition show smooth variation, being lower than 0.05 TECU/min. Generally, ROTI > 0.5 TECU/min indicates the presence of ionospheric irregularities at scale length of a few kilometers [27]. Note that there are few abnormal ROTIs being larger than 0.3 TECU/min under quiet condition. The abnormal results can be caused by many factors such as multipath. When satellite signals pass through the ionospheric irregularities, they would encounter signal attenuation, even sometimes complete loss of lock [28]. Hence, the degradation of PPP during the geomagnetic storm period should be attributed to ionospheric irregularities. The detailed relationship between ionospheric irregularities and degradation of PPP over the global scale are shown below.

**Figure 6:**
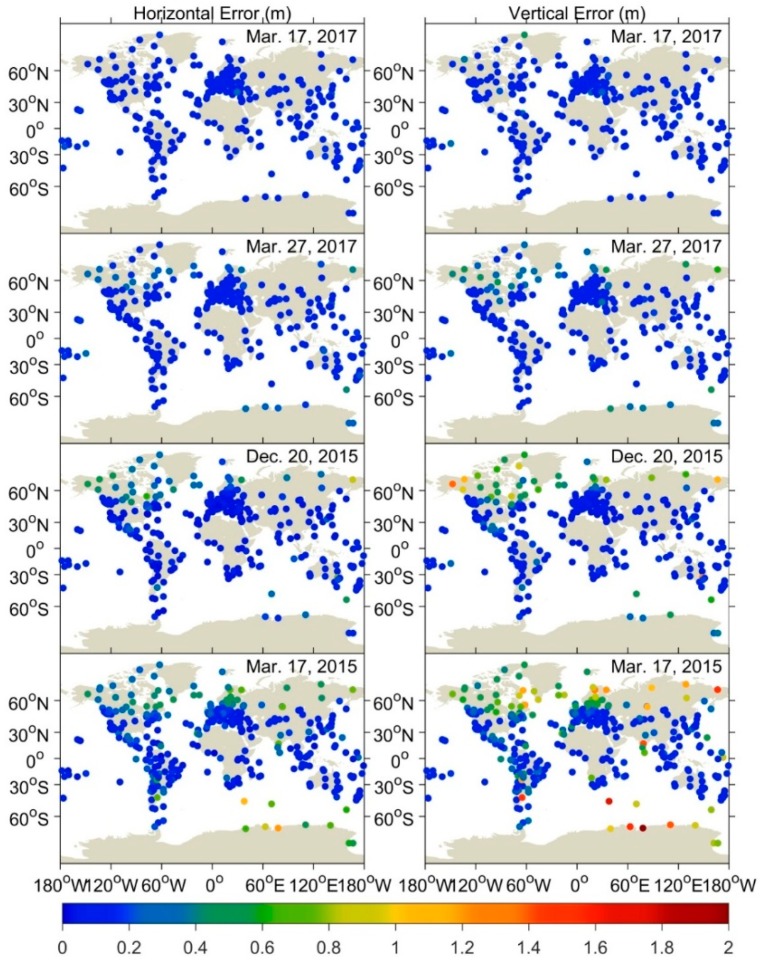
RMS statistics of DF PPP for worldwide GPS stations during the quiet, moderate, intense, and super storm period.

Figure 6 presents the RMS statistics of horizontal and vertical errors of GPS DF PPP for all IGS stations over the global scale for each storm day as well as the quiet day. Since the first two hours (0:00–2:00 UTC) are in the state of convergence in PPP solution, the RMS is calculated based on positioning errors in the period of 2:00–24:00 UTC for each day. The top two panels clearly show that under normal ionospheric condition positioning accuracies of GPS DF PPP in the horizontal and vertical components are better than 0.15 m and 0.2 m. During the moderate geomagnetic storm, some stations located in the high-latitude, e.g. North America region, experience the positioning quality deterioration as their RMS can reach around 0.5 m. With the increase of storm intensity, more and more stations are affected by the ionospheric disturbances, which can be obviously seen in the third and fourth panels. In the vertical component, stations with RMS > 0.5 m account for 1.7%, 7.0%, and 14.6% during moderate, intense, and super storms period, respectively. In addition, there are 19 stations with RMS > 1.0 m during the super storm of 17 March 2015.

**Figure 7:**
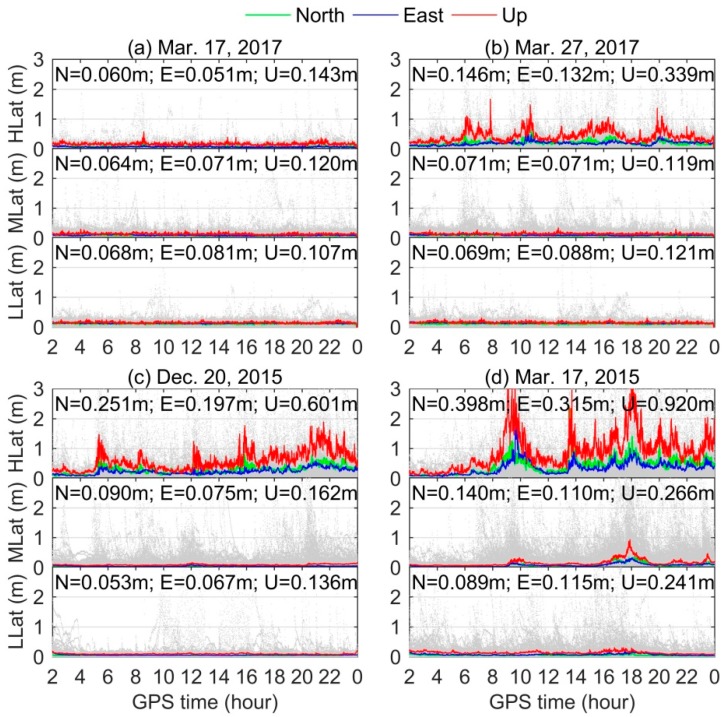
The 90% confidence level of time series of DF PPP results for all stations located at high-, mid-, and low-latitude regions on: (**a**) 17 March 2017 as the quiet day reference; (**b**) 27 March 2017 as the moderate storm; (**c**) 20 December 2015 as the intense storm; and (**d**) 17 March 2015 as the super storm, respectively. The background gray scatters are the absolute values of positioning results for each IGS station.

The 90% confidence level of time series of positioning errors of DF PPP over all stations located at high-latitude (60°–90° N/S), mid-latitude (30°–60° N/S), and low-latitude (0°–30° N/S) regions during three typical storms are presented in Figure 7. The background gray scatters are the positioning results for all IGS stations.For clarity, the first two hours results in the state of convergence are excluded in Figure 7. The corresponding RMS statistics in the north, east, and up components are also given in the figure. The three-dimensional (3D) RMS statistics for all stations are presented in Table 3. During geomagnetic storms, it can be clearly seen that more serious degradation of DF PPP is found in the high-latitude region compared with other two latitude regions. The degradations of mid- and low-latitude regions are comparable. For high-latitude region, the 3D RMSs during moderate, intense, and super storms are 0.393 m, 0.680 m, and 1.051 m, respectively, while under quiet condition is only 0.163 m (see Table 3). The performances of DF PPP for mid- and low-latitude regions are not very influenced by the moderate and intense storms, but few stations show major degradation during the super storm period.

**Figure 8:**
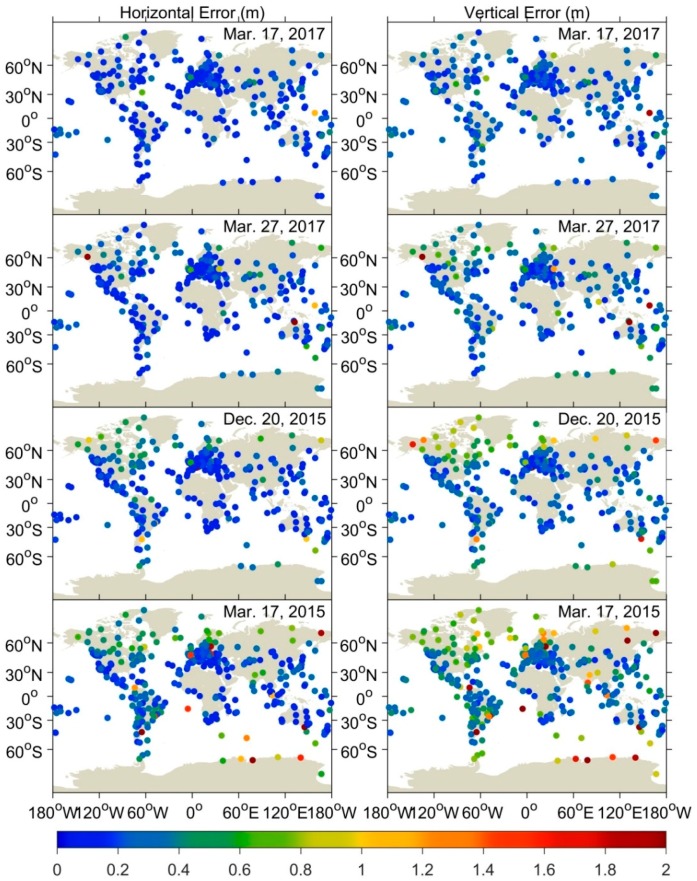
RMS statistics of SF PPP for worldwide GPS stations during the quiet, moderate, intense, and super storm period.

**Figure 9:**
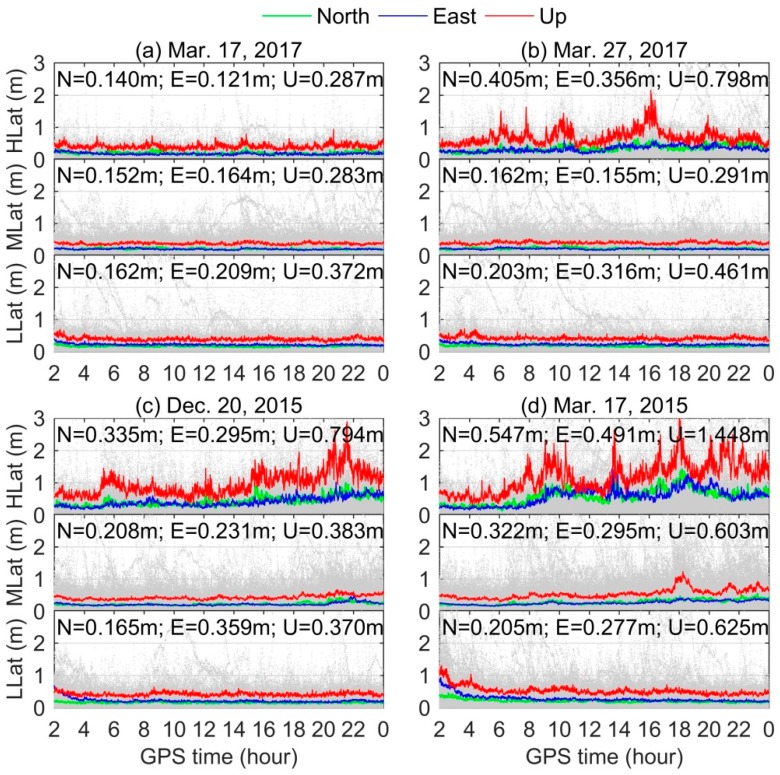
The 90% confidence level of time series of SF PPP results for all stations located at high-, mid-, and low-latitude regions on: (**a**) 17 March 2017 as the quiet day reference; (**b**) 27 March 2017 as the moderate storm; (**c**) 20 December 2015 as the intense storm; and (**d**) 17 March 2015 as the super storm, respectively. The background gray scatters are the absolute values of positioning results for each IGS station.

The corresponding results of GPS SF PPP are shown in Figure 8 and Figure 9. Similar to DF PPP, the degree of deterioration for SF PPP mainly depends on the intensity of geomagnetic storm. Due to the lack of L2 observations, the performance of SF PPP is inferior to that of DF PPP regardless of quiet or disturbed condition. Under quiet condition, the 3D RMS of SF PPP are 0.341 m, 0.361 m, and 0.456 m in high-, mid-, and low-latitude regions (see Table 3), which is consistent with the previous study [19]. During geomagnetic storms periods, it can be found that stations experiencing degraded positioning of SF PPP are mainly distributed in high-latitude region. Their 3D RMSs during moderate, intense, and super storms are 0.963 m, 0.911 m, and 1.624 m (see Table 3). Note that, during a super storm period, the degradation of SF PPP can also be clearly seen in mid- and low-latitude regions. Nava et al. [29] pointed out that, during the super storm period on 17 March 2015, the extreme ionospheric conditions have been observed in mid- and low-latitudes. Therefore, the deteriorated ionospheric correction caused by large gradients of ionosphere under super storm condition should be treated as the main reason for the degradation of positioning in mid- and low-latitude regions. It is easy to understand that, under severe ionospheric condition, ionospheric correction is a more serious challenge in SF solution compared with that in DF solution.

**Figure 10:**
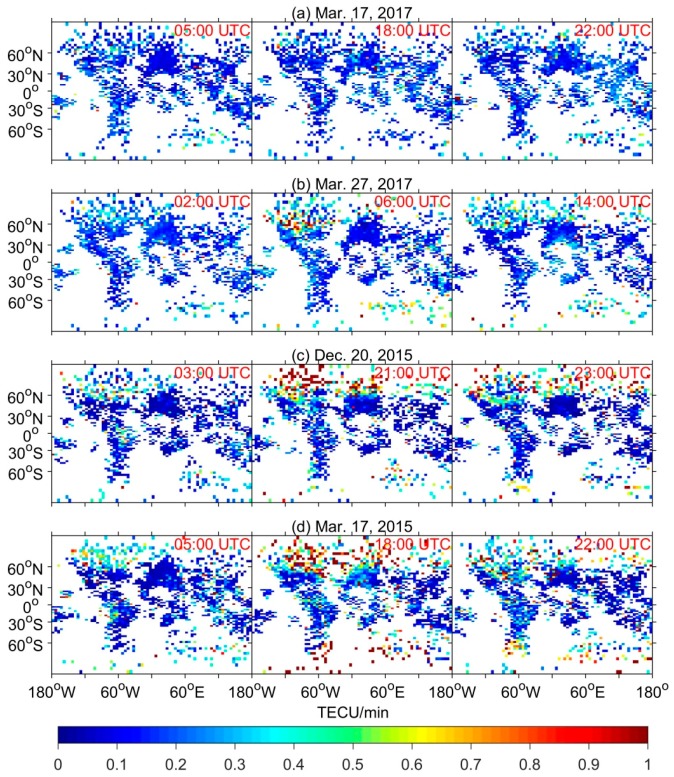
Global ROTI maps of the three perturbation times (i.e., the initial phase, storm development, and deep main phase) on: (**a**) 17 March 2017 as the quiet day reference; (**b**) 27 March 2017 as the moderate storm; (**c**) 20 December 2015 as the intense storm; and (**d**) 17 March 2015 as the super storm, respectively.

From the above analysis, it is found that the degraded performance of GPS DF and SF PPP should be attributed to the complex ionospheric conditions caused by geomagnetic storms. Therefore, it is necessary to show the specific ROTI data to further analyze the ionospheric disturbances during different intensities storms. Figure 10 presents the global ROTI maps derived from entire IGS network in the three perturbation periods (i.e., initial phase, storm development, and deep main phase) of each geomagnetic storm. The ROTI values are averaged in grid of 2.5° latitude and 5° longitude with a sliding window for each 5 min interval. As the GPS phase fluctuation index, ROTI can be used to monitor global activity of ionospheric irregularities [27,30]. From the topside panels, we can clearly observe that most ROTI values are smaller than 0.3 TECU/min, indicating no obvious irregularity occurrences on the quiet day of 17 March 2017. Consequently, the corresponding results of GPS PPP are in agreement with the expectations. In the case of the geomagnetic storms, the ROTI values would be different. During the initial phase period, as shown in the left three panels, the irregularities appeared at high-latitude of northern America sector as well as Antarctic sector where some ROTI values are larger than 0.5 TECU/min. Since this period is in the initial phase of the storms, the irregularities are not commonly seen in other sectors. Afterwards, the storms went into the main phase. The significant irregularities can be easily seen in high-latitude regions including North America, northern Europe as well as Antarctic region, and some ROTI values even can reach 1 TECU/min. Note that the stronger intensity of geomagnetic storms, the higher ROTI values and larger scale of irregularities are found in general. Consequently, more stations are affected by the irregularities (see Figure 6 and Figure 8). In addition, during the main phase period around 18:00 UTC of the super storm, the irregularities can also be found in the equatorial regions such as the India sector. During the deep main phase close to recovery phase, shown in the right three panels, the intensity of irregularities decreases gradually although the auroral activities were also active especially in the Antarctic and South America regions during the super storm.

**Figure 11:**
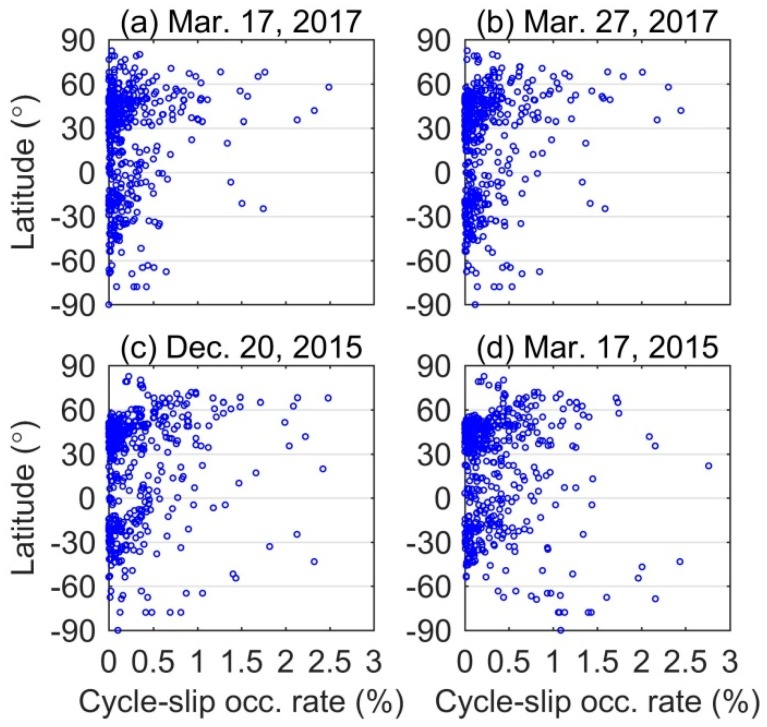
Distribution of cycle-slip occurrence rate against geomagnetic latitude for all IGS stations statistics on: (**a**) 17 March 2017 as the quiet day reference; (**b**) 27 March 2017 as the moderate storm; (**c**) 20 December 2015 as the intense storm; and (**d**) 17 March 2015 as the super storm, respectively.

As discussed before, the ionospheric irregularities caused by geomagnetic storms possibly lead to the occurrence of cycle-slip and even loss of signal tracking [31]. The frequent cycle-slip occurrence can result in degradation of GNSS positioning. To further analyze the characteristics of cycle-slip occurrence under different intensities storms, Figure 11 gives the scatters of cycle-slip occurrence rate against geographical latitude derived from all IGS stations data during the quiet, moderate, intense, and super storm periods. In this study, the Melbourne-Wübbena wide-land combination and rate of geometry-free combination proposed by Luo et al. [9] are jointly used to detect cycle-slip. Note that cycle-slip occurrence rate is calculated as the total number of cycle-slips divided by total available measurements.

From the topside two panels, we can see that most scatters are within the range of 0–0.5%. Compared with the South Pole, more cycle-slips are detected in the North Pole, which is consistent with the results reported by Astafyeva et al. [32]. From the bottom two panels, we can find that the scatters are more discrete mainly ranging from 0% to 1%. That means many cycle-slips occurred under intense and super storms conditions. Meanwhile, although there are fewer stations located in the South Pole, the cycle-slip occurrence rate can reach around 2% under super storm condition. That implies larger scale irregularities induced by super storm resulted in cycle-slip occurrence in the two poles. The statistics show that the mean values of cycle-slip occurrence rate are 0.23%, 0.26%, 0.36%, and 0.39% for quiet, moderate, intense, and super storm days, respectively. Compared with quiet days, the increased cycle-slips occurrence percentages are 13.04%, 56.52%, and 69.57% during moderate, intense, and super storm days. From the above analysis, we conclude that the degraded performance of GPS PPP under geomagnetic storm conditions should be attributed to two factors: (1) the deteriorated ionospheric correction under severe ionospheric conditions; and (2) the contaminated satellite measurements, such as serious signal attenuation and frequent cycle-slip occurrence, when satellites signals pass through the ionospheric irregularities.

## 4. Discussion

This study presents a comprehensive investigation of the performance of GPS DF and SF PPP under three typical geomagnetic storms, using a large data set collected from about 500 IGS stations, during solar cycle 24 from 1 January 2011 to 31 December 2017. The geomagnetic storms are the moderate storm on 27 March 2017 with Dst_min_ = −74 nT, intense storm on 20 December 2015 with Dst_min_ = −155 nT, and super storm on 17 March 2015 with Dst_min_ = −233 nT.

Under geomagnetic storms conditions, the time series of positioning results derived from high-latitude stations data for GPS DF and SF PPP vary significantly and the range can reach several meters in the horizontal and vertical components. This is consistent with the previous investigations based on RTK [13,14,15,16] and PPP techniques [17]. With the increase of storm intensity, more and more stations experienced the degradation of positioning with most located at high-latitude regions. It is well known that the high-latitude region has strong coupling among the interplanetary medium, the magnetosphere, the thermosphere, and the ionosphere [29]. During geomagnetic storms, abundant energetic particles precipitate into high-latitude region, and heat the upper atmosphere in the form of Joule heating and Lorentz forces, resulting in the steep ionospheric density gradients and irregularities [5,33]. Our global ROTI maps clearly show that the ionospheric irregularities are mainly distributed in high-latitude regions including North America, northern Europe and Antarctic region during the main phase period of geomagnetic storms (see Figure 10). As a result, most stations located at high-latitude region experienced the degradation of positioning.

To GNSS users, adverse effects caused by the geomagnetic storm should be carefully mitigated to ensure the reliable positioning requirement. The mitigation strategies can be summarized as three parts: improvement of the success rate of cycle-slip detection and correction during the data processing [34]; consideration of the second- or high-order ionospheric delays in ionospheric corrections [15,35]; and combination of other GNSS system satellites to improve the weak satellite geometries [36]. The frequent cycle-slip occurrence during geomagnetic storms has been demonstrated in Figure 11. Since most approaches of cycle-slip detection and correction lack proper handling of ionospheric delay variations, a geometry-based approach with rigorous handling of the ionosphere was presented by Banville and Langley [34]. After applying this method to GNSS data collected in northern Canada, some kinematic PPP results showed a dm-level improvement. However, the increased measurement noise associated with an active ionosphere still makes cycle-slip detection and correction difficulty. Under geomagnetic storm conditions, the first-order ionospheric delays can reach about 30 m and the second-order delays are very small but with about 3 mm magnitude [35,37]. Therefore, consideration of high-order ionospheric delays can only contribute to an improvement of mm-level in GNSS PPP. The integration of multi-constellation GNSS can significantly increase the number of satellites, thereby increasing the measurement redundancy and improving the satellite geometry. However, the positioning errors of GPS/GLONASS PPP in the height component can also reach around 1 m during the geomagnetic storm period on 13 May 2015 [36]. From the above analysis, we can conclude that aforementioned strategies may also be influenced by severe geomagnetic storm conditions. More reliable mitigation strategies require further investigation.

## 5. Conclusions

A comprehensive investigation of geomagnetic storms effects on GPS PPP has been presented in our study. Results show that under normal condition without geomagnetic storms occurrence GPS DF PPP accuracy of most IGS stations is better than 0.15 m and 0.2 m in the horizontal and vertical components, respectively. The degradation performance of DF PPP can be easily found in geomagnetic storms conditions especially at high-latitude region. The 3D RMS at this region based on all stations’ results during moderate, intense, and super storms are 0.393 m, 0.680 m, and 1.051 m, respectively, while that value is only 0.163 m under quiet condition. In addition, our results show that stronger intensity storms generally cause more significant degradation of positioning. The statistics indicate that the number of stations with RMS > 0.5 m accounts for 1.7%, 7.0%, and 14.6% during moderate, intense, and super storm periods, respectively. Besides, there are 19 stations with RMS > 1.0 m during the super storm of 17 March 2015. Compared with DF PPP, the performance of GPS SF PPP is inferior regardless of quiet or disturbed condition. Under quiet condition, GPS SF PPP accuracy of most IGS stations is around 0.35 m and 0.6 m in the horizontal and vertical components. Under disturbed condition, the 3D RMS of SF PPP during moderate, intense, and super storms can reach 0.963 m, 0.910 m, and 1.624 m at high-latitude region, respectively. Different from DF PPP, more serious degradation of SF PPP can also be clearly found at mid- and low-latitude regions, especially for the super storm condition.

During geomagnetic storm periods, the degraded performance of GPS positioning regardless of DF or SF PPP should be attributed to the increased ionosphere disturbances. The strong response between positioning errors and ionospheric irregularities index ROTI has been confirmed in this study based on single station data collected at high-latitude. Moreover, the global ROTI maps further indicated the strong correlation between the degradation positioning and ionospheric irregularities. The ionospheric disturbances can not only lead to the deteriorated ionospheric correction but also to frequent cycle-slip occurrence. Statistical results show that the increased cycle-slip occurrence percentages are 13.04%, 56.52%, and 69.57% under moderate, intense, and super storms conditions, respectively, compared with those of quiet day. Note that this study only focuses on GPS PPP performance analysis under different geomagnetic storm conditions. The corresponding multi-GNSS PPP analysis requires further investigation.

## Figures and Tables

**Table 1:** Summary of data processing models and strategies for GPS DF and SF PPP.

Item	Models and Strategies
Observations	Raw code and carrier phase observations from L1/L2 signal
Observation weighting	Elevation-dependent weight
Sampling rate	30 s
Elevation angle cutoff	10°
Satellite orbit	Fixed using the products from IGS
Satellite clock	Fixed using the products from IGS
Phase wind-up effect	Corrected
Phase center offset	igs08.atx
Phase center variation	igs08.atx
Ionospheric delay (DF/SF)	Piece-wise for polynomials in 5 min and random walk for temporal variation, GIM from CODE as a priori information constraint [25]
Tropospheric delay	Prior model as Hopfield model [26] with remaining estimated as a random walk process (5 mm/√h)
Receiver coordinate	Estimated and modeled as a random walk process [24]
Differential code biases (DCB)	Corrected using the products from CODE

**Table 2:** The maximum (MAX) values and root mean square (RMS) statistics of GPS DF and SF PPP during the quiet, moderate, intense, and super storms period (Unit: m).

		DF PPP				SF PPP			
		North	East	Up	3D	North	East	Up	3D
MAX	17 March 2017 (Quiet)	−0.172	0.258	−0.572	0.599	0.284	0.248	−1.050	1.077
	27 March 2017 (Moderate)	−1.154	−1.666	−4.153	4.247	−1.069	0.953	−3.691	3.742
	20 December 2015 (Intense)	−1.562	−0.880	−4.292	4.601	1.389	−1.431	−3.045	3.333
	17 March 2015 (Super)	12.293	3.639	22.009	25.215	6.804	2.210	16.727	18.101
RMS	17 March 2017 (Quiet)	0.040	0.068	0.106	0.132	0.083	0.085	0.179	0.215
	27 March 2017 (Moderate)	0.151	0.119	0.310	0.365	0.195	0.184	0.586	0.644
	20 December 2015 (Intense)	0.225	0.148	0.609	0.703	0.314	0.372	0.602	0.774
	17 March 2015 (Super)	0.671	0.221	1.224	1.413	0.609	0.296	1.126	1.314

**Table 3:** The 3D RMS statistics of GPS DF and SF PPP for all stations in high-, mid-, and low-latitudes during the quiet, moderate, intense, and super storms period (Unit: m).

	DF PPP				SF PPP			
Latitude	Quiet	Moderate	Intense	Super	Quiet	Moderate	Intense	Super
High-	0.163	0.393	0.680	1.051	0.341	0.963	0.911	1.624
Mid-	0.153	0.156	0.200	0.320	0.361	0.368	0.493	0.745
Low-	0.151	0.165	0.160	0.281	0.456	0.595	0.542	0.714

## References

[1] Gonzalez W.D., Joselyn J.A., Kamide Y., Kroehl H.W., Rostoker G., Tsurutani B.T., Vasyliunas V.M. (1994). What is a geomagnetic storm?. J. Geophys. Res. Space Phys..

[2] Srivastava N., Venkatakrishnan P. (2004). Solar and interplanetary sources of major geomagnetic storms during 1996-2002. J. Geophys. Res. Space Phys..

[3] Rama Rao P.V.S., Gopi Krishna S., Vara Prasad J., Prasad S.N.V.S., Prasad D.S.V.V.D., Niranjan K. (2009). Geomagnetic storm effects on GPS based navigation. Ann. Geophys..

[4] Skone S. (2001). The impact of magnetic storms on GPS receiver performance. J. Geod..

[5] Cherniak I., Zakharenkova I. (2015). Dependence of the high-latitude plasma irregularities on the auroral activity indices: A case study of 17 March 2015 geomagnetic storm. Earth Planets Space.

[6] Van Der Meeren C., Oksavik K., Lorentzen D.A., Rietveld M.T., Clausen L.B.N. (2015). Severe and localized GNSS scintillation at the poleward edge of the nightside auroral oval during intense substorm aurora. J. Geophys. Res. Space Phys..

[7] Carter B.A., Yizengaw E., Pradipta R., Retterer J.M., Groves K., Valladares C., Caton R., Bridgwood C., Norman R., Zhang K. (2016). Global equatorial plasma bubble occurrence during the 2015 St. Patrick’s Day storm. J. Geophys. Res. A Space Phys..

[8] Li G., Ning B., Zhao B., Liu L., Liu J.Y., Yumoto K. (2008). Effects of geomagnetic storm on GPS ionospheric scintillations at Sanya. J. Atmos. Sol. Terr. Phys..

[9] Luo X., Liu Z., Lou Y., Gu S., Chen B. (2017). A study of Multi-GNSS ionospheric scintillation and cycle-slip over Hong Kong region for moderate solar flux conditions. Adv. Space Res..

[10] Luo X., Lou Y., Xiao Q., Gu S., Chen B., Liu Z. (2018). Investigation of ionospheric scintillation effects on BDS precise point positioning at low-latitude regions. GPS Solut..

[11] Marques H.A., Marques H.A.S., Aquino M., Veettil S.V., Monico J.F.G. (2018). Accuracy assessment of Precise Point Positioning with multi-constellation GNSS data under ionospheric scintillation effects. J. Space Weather Space Clim..

[12] Rizos C. (2002). Network RTK Research and Implementation: A Geodetic Perspective. J. Glob. Position Syst..

[13] Jacobsen K.S., Schäfer S. (2012). Observed effects of a geomagnetic storm on an RTK positioning network at high latitudes. J. Space Weather Space Clim..

[14] Wielgosz P., Kashani I., Grejner-Brzezinska D. (2005). Analysis of long-range network RTK during a severe ionospheric storm. J. Geod..

[15] Bergeot N., Bruyninx C., Defraigne P., Pireaux S., Legrand J., Pottiaux E., Baire Q. (2011). Impact of the Halloween 2003 ionospheric storm on kinematic GPS positioning in Europe. GPS Solut..

[16] Lejeune S., Wautelet G., Warnant R. (2012). Ionospheric effects on relative positioning within a dense GPS network. GPS Solut..

[17] Jacobsen K.S., Andalsvik Y.L. (2016). Overview of the 2015 St. Patrick’s day storm and its consequences for RTK and PPP positioning in Norway. J. Space Weather Space Clim..

[18] Zumberge J.F., Heftin M.B., Jefferson D., Watkins M.M., Webb F.H. (1997). Precise point positioning for the efficient and robust analysis of GPS data from large networks. J. Geophys. Res..

[19] Lou Y., Zheng F., Gu S., Wang C., Guo H., Feng Y. (2016). Multi-GNSS precise point positioning with raw single-frequency and dual-frequency measurement models. GPS Solut..

[20] Echer E., Gonzalez W.D., Tsurutani B.T., Gonzalez A.L.C. (2008). Interplanetary conditions causing intense geomagnetic storms (Dst ≤ -100 nT) during solar cycle 23 (1996-2006). J. Geophys. Res. Space Phys..

[21] Kamide Y., Yokoyama N., Gonzalez W., Tsurutani B.T., Daglis I.A., Brekke A., Masuda S. (1998). Two-step development of geomagnetic storms. J. Geophys. Res..

[22] Echer E., Tsurutani B.T., Gonzalez W.D. (2013). Interplanetary origins of moderate (−100 nT <Dst ≤ −50 nT) geomagnetic storms during solar cycle 23 (1996–2008). J. Geophys. Res. Space Phys..

[23] Zhang J., Richardson I.G., Webb D.F., Gopalswamy N., Hutrunen E., Kasper J.C., Nitta N.V., Poomvises W., Thompson B.J., Wu C.C. (2007). Solar and interplanetary sources of major geomagnetic storms (Dst ≤ −100 nT) during 1996-2005. J. Geophys. Res. Space Phys..

[24] Gu S., Shi C., Lou Y., Liu J. (2015). Ionospheric effects in uncalibrated phase delay estimation and ambiguity-fixed PPP based on raw observable model. J. Geod..

[25] Shi C., Gu S., Lou Y., Ge M. (2012). An improved approach to model ionospheric delays for single-frequency Precise Point Positioning. Adv. Space Res..

[26] Hopfield H.S. (1971). Tropospheric effect on electromagnetically measured range: Prediction from surface weather data. Radio Sci..

[27] Ma G., Maruyama T. (2006). A super bubble detected by dense GPS network at east Asian longitudes. Geophys. Res. Lett..

[28] Xiong C., Stolle C., Lühr H. (2016). The Swarm satellite loss of GPS signal and its relation to ionospheric plasma irregularities. Space Weather.

[29] Nava B., Rodríguez-Zuluaga J., Alazo-Cuartas K., Kashcheyev A., Migoya-Orué Y., Radicella S.M., Amory-Mazaudier C., Fleury R. (2016). Middle- and low-latitude ionospheric response to 2015 St.Patrick’s Day. J. Geophys. Res. Space Phys..

[30] Pi X., Mannucci A.J., Lindqwister U.J., Ho C.M. (1997). Monitoring of global ionospheric irregularities using the worldwide GPS Network. Geophys. Res. Lett..

[31] Zakharov V.I., Yasyukevich Y.V., Titova M.A. (2016). Effect of magnetic storms and substorms on GPS slips at high latitudes. Cosm. Res..

[32] Astafyeva E., Yasyukevich Y., Maksikov A., Zhivetiev I. (2014). Geomagnetic storms, super-storms, and their impacts on GPS-based navigation systems. Space Weather.

[33] Yao Y., Liu L., Kong J., Zhai C. (2016). Analysis of the global ionospheric disturbances of the March 2015 great storm. J. Geophys. Res. Space Phys..

[34] Banville S., Langley R.B. (2013). Mitigating the impact of ionospheric cycle slips in GNSS observations. J. Geod..

[35] Hoque M.M., Jakowski N. (2007). Higher order ionospheric effects in precise GNSS positioning. J. Geod..

[36] Juan J.M., Sanz J., González-Casado G., Rovira-Garcia A., Camps A., Riba J., Barbosa J., Blanch E., Altadill D., Orus R. (2018). Feasibility of precise navigation in high and low latitude regions under scintillation conditions. J. Space Weather Space Clim..

[37] Pireaux S., Defraigne P., Wauters L., Bergeot N., Baire Q., Bruyninx C. (2010). Higher-order ionospheric effects in GPS time and frequency transfer. GPS Solut..

